# Adenosine Receptors as Potential Therapeutic Analgesic Targets

**DOI:** 10.3390/ijms241713160

**Published:** 2023-08-24

**Authors:** Mansour Haddad, Federica Cherchi, Mohammad Alsalem, Yousef M. Al-saraireh, Saba Madae’en

**Affiliations:** 1 Faculty of Pharmacy, Yarmouk University, Irbid 21163, Jordan; 2 Department of Neuroscience, Psychology, Drug Research and Child Health (NEUROFARBA), Section of Pharmacology and Toxicology, University of Florence, 50139 Florence, Italy; federica.cherchi@unifi.it; 3 School of Medicine, The University of Jordan, Amman 11942, Jordan; m_alsalem@ju.edu.jo; 4 Department of Pharmacology, Faculty of Medicine, Mutah University, P.O. Box 7, Al-Karak 61710, Jordan; yousef.sar@mutah.edu.jo; 5 Department of Clinical Pharmacy and Pharmacy Practice, Faculty of Pharmaceutical Sciences, The Hashemite University, Zarqa 13133, Jordan; saba@hu.edu.jo

**Keywords:** GPCR, adenosine, pain, inflammation, purinergic signaling

## Abstract

Pain represents an international burden and a major socio-economic public health problem. New findings, detailed in this review, suggest that adenosine plays a significant role in neuropathic and inflammatory pain, by acting on its metabotropic adenosine receptors (A_1_AR, A_2A_AR, A_2B_AR, A_3_AR). Adenosine receptor ligands have a practical translational potential based on the favorable efficacy and safety profiles that emerged from clinical research on various agonists and antagonists for different pathologies. The present review collects the latest studies on selected adenosine receptor ligands in different pain models. Here, we also covered the many hypothesized pathways and the role of newly synthesized allosteric adenosine receptor modulators. This review aims to present a summary of recent research on adenosine receptors as prospective therapeutic targets for a range of pain-related disorders.

## 1. Introduction

### 1.1. Pain

Chronic pain is a global burden and a major socio-economic public health problem, profoundly limiting and adversely impacting the quality of life and promoting disability and unnecessary suffering [1]. An estimated 10–30% of people worldwide are affected, with a high societal impact [2,3]. Despite extensive investigational efforts, current treatment approaches (nonsteroidal anti-inflammatory drugs, opioids, antidepressants, or anticonvulsants) for chronic pain are ineffective, inadequate, and/or have intolerable side effects [4,5,6,7]. New drugs and therapies are required because a safe and effective treatment for chronic pain has not yet been developed. Pharmaceutical research focuses on effective analgesic drugs, with G-protein-coupled receptors (GPCRs) being a key focus in pain-relieving medication development [8,9,10]. Among them, adenosine metabotropic receptors (AR) modulators are increasingly being implicated in the antinociceptive and/or antihyperalgesic effects seen in various chronic pain models [2,11,12,13]. This review aims to describe the most recent developments in our knowledge of the function of ARs in pain. We will review and analyze the preclinical experimental studies that examined the function and mode of action of each receptor subtype in the modulation of acute and chronic pain.

### 1.2. Role of Adenosine in Inflammation and Pain

New findings suggest that adenosinergic modulators have substantial therapeutic potential in chronic diseases, particularly, in inflammation dysregulation situations [14]. In neuropathic and inflammatory pain models, adenosine controls neuronal and non-neuronal cell functions [15], modulates primary afferent transmission, and prevents pain behavior. Furthermore, adenosine exerts multiple effects on the transmission of pain in peripheral and spinal areas and plays an important role in CNS pain processing by regulating excitatory neurotransmission, sustaining neuronal signal transmission, and regulating glial activation and proliferation [7,15,16,17,18]. Adenosine is an endogenous inflammation modulator that influences almost all physiological and pathophysiological functions [19,20]. Indeed, the adenosinergic system represents an outstanding pharmacological target for pain management, in which inflammation represents a pathogenetic mechanism [11,20]. Indeed, ARs mainly serve as anti-inflammatory receptors since they have an important role in modulating cytokine release and expression as well as in oxidation damage [11]. Adenosine signaling also stimulates the release of neurotransmitters in the brain [21] and contributes to the antinociceptive features of norepinephrine, opioids, caffeine, tricyclic antidepressants, 5-hydroxytryptamine, and transcutaneous electrical nerve stimulation [16]. Adenosine receptor modulators administered spinally or systemically cause antinociception in a large number of studies [12,22,23,24,25], both in animal models [21,26] and in clinical studies [27,28].

The role of AR activation in inflammatory pain models has been recently investigated [13]. Inflammation can lead to altered pain perception and is mediated by proinflammatory chemokines, reactive oxygen species, and various other mediators and secondary messengers [29]. During this event, also extracellular adenosine concentrations are heightened from multiple sources, including vesicular release and its precursor ATP. Thus, the nonspecific or mechanism-based release of nucleotides, which are later metabolized to adenosine, can indirectly increase the concentrations of adenosine [15]. Adenosine can modulate intrinsic neuronal transmission by increasing K^+^ conductivity and, presynaptically, at sensory nerve endings to prevent the release of substance P and, potentially, glutamate [30,31]. The effects of adenosine on inflammation regulation support the therapeutic application of selective agonists or antagonists capable of activating or inactivating ARs in numerous diseases and conditions, notoriously inflammatory pain.

An initial investigation highlighted the effectiveness of intrathecal (i.t.) adenosine analogs in lowering hypersensitive symptoms [32]. Further research verified their substantial action in nerve damage models, and it has been underlined that adenosine systems may serve as a target in neuropathic pain syndromes. The spinal infusion of adenosine relieved mechanical allodynia after nerve damage, but not in healthy animals [21]; this effectiveness was remarkably long-lasting (i.e., to 24 h; [33]). In addition, when adenosine is administered in an inflamed area, leukocyte rise, activation, and adhesion are reduced [34] (Table 1).

### 1.3. Adenosine Metabolism

Adenosine is a ubiquitous but short-lived endogenous modulator [14] whose effects are mostly exerted by the activation of its four G protein-coupled P1 receptor subtypes: A_1_, A_2A_, A_2B_, and A_3_ ARs [47,66,73,74]. The effects of each AR depend on the extracellular adenosine concentrations, which are controlled by different intra- and extra-cellular enzymes involved in its synthesis and degradation, as well as by uptake processes at the membrane level. Using this sophisticated system, the cells can adjust purinergic signaling in response to changes in the health state of a tissue [18]. The ectonucleotidase CD39 converts ATP/ADP to AMP, and subsequently, CD73 converts AMP to adenosine. This sequence is the primary source of adenosine in the extracellular space [75]. Adenosine, in turn, is removed by enzymes that are specifically designed to metabolize it, such as adenosine deaminase (ADA), which converts extracellular adenosine to inosine, and adenosine kinase, which phosphorylates adenosine to form AMP [76]. These latter two intracellular enzymes reduce extracellular adenosine to attenuate AR signaling because these two pools are linked through equilibrative nucleoside transporters (ENTs) and concentrative nucleoside transporters (CNTs) on the cell membrane [77]. Typical extracellular endogenous adenosine concentrations during normal conditions in tissues and organs are in the nanomolar range [78]. However, adenosine is dramatically elevated in the vicinity of damaged cells following trauma or injury, particularly under hypoxic/ischemic insults [79,80], where it can reach micromolar concentrations and consequently activate all AR subtypes [18].

## 2. Adenosine Receptors

**Figure 1:**
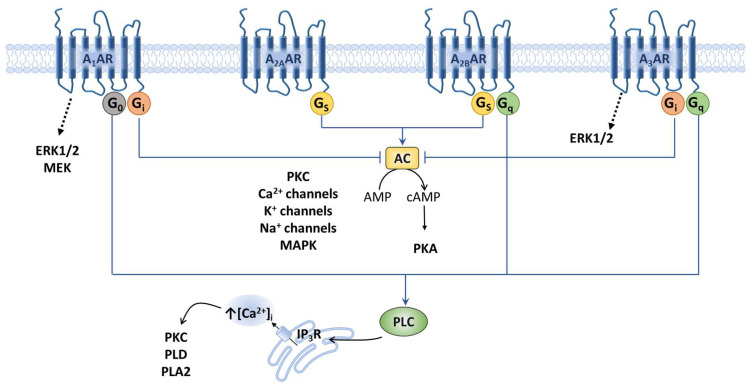
**Adenosine receptors and their main signaling pathways.** Schematic representation of G-coupled A_1_A, A_2A_, A_2B_, and A_3_A receptor subtypes (A_1_AR, A_2A_AR, A_2B_AR, and A_3_AR) activated by extracellular adenosine and the main intracellular pathways involved. A_2_AR and A_2B_AR receptors are coupled to Gs protein, which leads to adenylyl cyclase (AC) activation and cyclic AMP (cAMP) increase. On the other hand, A_1_AR and A_3_AR are coupled to Gi protein, which inhibits AC and reduces cAMP. In some districts, A_2B_AR and A_3_AR receptors are also coupled to Gq protein, and A_1_AR is coupled to Go, which stimulates Ca^2+^ release from intracellular stores. Moreover, all receptors are coupled to mitogen-activated protein kinase (MAPK) pathways, including extracellular signal-regulated kinase 1 (ERK1), ERK2, p38, and MAPK.

Various studies probed which ARs, when either activated or blocked, can contribute to an antinociceptive effect. The receptor subtypes differ in the G subunit to which they bind: A_1_AR and A_3_AR are preferentially coupled to Gi proteins, which means that they inhibit the activity of adenylyl cyclase (AC); while A_2A_AR and A_2B_AR stimulate AC via Gs proteins, resulting in increased cyclic adenosine monophosphate (cAMP) levels [19,47,73]. Adenosine can have various impacts on peripheral pain signals (i.e., normal tissue, inflammation, or following nerve injury), depending on the implicated receptor subtype, its location, and the tissue circumstances (Figure 1).

Increases in cAMP at the sensory nerve terminal and the activation of PKA have been shown to induce hyperalgesia [81,82]. In this context, a recent study shows that, following partial sciatic nerve ligation in rats, cAMP inhibition reduces the behavioral signs of neuropathic pain and the phosphorylation of the cAMP response element binding (CREB) protein in the spinal cord [83]. Researchers have been able to better understand the role of these receptors in pain signaling by monitoring how gene deletions alter thresholds to various types of pain stimulation since the development of A_1_, A_2A_, A_2B_, and A_3_AR-deficient mouse strains [47].

### 2.1. Adenosine A_1_ Receptors

The G_i/o_ protein family includes A_1_AR, and its activation causes a majority of the biological effects mostly by inhibiting the second messenger cAMP [17,84,85]. In addition, when activated, the G protein beta and gamma subunits stimulate phospholipase C [86], which cleaves phosphatidylinositol-4,5-bisphosphate [87] into diacylglycerol (DAG) and inositol-1,4,5-trisphosphate (IP3), thereby increasing the Ca^2+^ levels. Additionally, elevated intracellular Ca^2+^ levels can trigger the activity of several enzymes, including protein kinase C [88], phospholipase D (PLD), phospholipase A2 (PLA2), and others. The PI3K and MAPK signaling pathways, specifically, ERK1/2 and MEK, can be activated by A_1_AR activation, which induces alterations in gene expression and the activation of glial cells [51,88,89].

A_1_AR activation can elicit both pro-inflammatory and anti-inflammatory responses. According to the literature, adenosine can serve as an analgesic by binding to A_1_ARs [41] and as an inducer of an anti-inflammatory response by binding to A_2A_ARs [90] in the early phases of inflammation. Activated spinal A_1_AR prevents sensory transmission by inhibiting the slow ventral root potential that is connected to nociception through C-fibers.

Additionally, i.t. adenosine enhances noradrenaline release in in vivo models through A_1_AR activation, but not under normal conditions [40,41], and its antiallodynic effects rely probably on spinal adrenergic processes [21]. Substances that cause an increase in endogenous adenosine concentrations, such as inhibitors of adenosine metabolism, can activate spinal adenosine A_1_ARs. As a result, increased antinociception is produced by the intravenous administration of prototype nucleoside inhibitors of adenosine kinase [30,90]). After systemic administration, a group of novel nucleoside and non-nucleoside adenosine kinase inhibitors (A-134974, A-286501, ABT-702) were shown to reduce inflammatory and neuropathic pain [31,91,92,93]. The adenosine concentration in the spinal cord was raised as a result of adenosine metabolism inhibition, which resulted in the activation of A_1_ARs [94,95]. While the intracerebroventricular (supraspinal) and intraplantar (peripheral) activities of adenosine kinase inhibitors were less effective compared to i.t. administration in neuropathic pain models, the antinociceptive effects of these drugs largely occurred at spinal locations [93]. Accordingly, peripheral locations were not involved in analgesic effects in an inflammatory condition [91].

A_1_AR activation is necessary for the majority of antinociceptive effects of adenosine [35,36]. A_1_ARs have recently been discovered to be present on intrinsic neurons and in laminae I and II of the dorsal horn of the spinal cord [17,37,38,96,97]. At the II lamina level, numerous afferent sensory nerves interact with postsynaptic neurons. In addition, A_1_ARs are localized in the descending projection within the posterior horn [38]. Presynaptic receptors could be present, since dorsal root ligation increased A_1_AR immunoreactivity on the side next to the dorsal root ganglion (DRG), despite rhizotomy failing to detect a significant presynaptic A_1_AR population on sensory neuron terminals [37,98]. Neuronal activity in the spinal cord and the DRGs is predominantly prevented by A_1_AR activation [25,42,43,99]. The ability of A_1_AR to regulate presynaptic excitatory transmission to substantia gelatinosa neurons in the spinal cord was connected to its ability to reduce pain [100]. According to DeLander et al. [101], the spinal cord is likely where adenosine-induced NMDA receptor inhibition also takes place, which lessens the plasticity and central sensitization mechanisms associated with chronic pain.

Additionally, multiple animal models of acute pain patterns showed that systemic administration of different A_1_AR agonists might have an analgesic effect [17,21,26,30,102,103]. The intracellular NO/cGMP/PKG/KATP signaling pathway is activated by peripheral adenosine A_1_AR, leading to reduced pain [35]. The A_1_AR agonists alleviate thermal, but not mechanical, allodynia induced by sciatic nerve damage. Since thermal hyperalgesia is mediated by C-fibers and mechanical allodynia by A-fibers, these results demonstrate that A_1_ARs are present in C- but not in A-fibers [15]. A_1_AR agonists, such as R-phenyl-isopropyl-adenosine (R-PIA) [104] and GR79236 [16], inhibit the mechanical allodynia induced by spinal nerve ligation in rats. GR79236 inhibits also carrageenan-induced inflammatory hyperalgesia [16], while R-PIA decreases the thermal pain threshold in rats with spinal cord damage [105]. The latter prevents formalin-induced pain and reduces hyperalgesia induced by PGE2 by activating A_1_AR [44,53]; however, it is to be noted that R-PIA is poorly selective for A_1_AR and can induce A_3_AR effects as well [106,107]. Another A_1_AR agonist, *N*^6^-cyclopentyladenosine (CPA), when administered to rats, prevented arthritis-induced pain and neuropathy-induced pain [46]. In particular, reports suggested that the i.t. administration of A_1_AR agonists caused analgesia in multiple animal models of acute pain, comprising tail flicking, tail immersion, hot plate, formalin, acetic acid, capsaicin models, and others [104,108,109]. Moreover, the analgesic effect induced by i.t. adenosine and the increase in thermal hyperalgesia in A_1_AR knockout mice were reversed [110]. In these animals, a lower pain threshold was found in hyperalgesia tests [12], and they also showed moderate hyperalgesia to heat stimulation [110].

Other mechanisms, such as the inhibition of glutamate release, are involved in A1AR-associated analgesic processes in chronic pain. As mentioned above, the antinociceptive impact of A_1_AR agonists has been ascribed to AC inhibition and the reduced generation of cAMP in sensory nerve endings [44,53,106,111]. Moreover, A_1_ARs inhibit calcitonin gene-related peptide release [16] and Ca^2+^ entry in sensory neurons when studied in vitro [106,107]. The primary mechanisms for spinal A_1_AR-mediated antinociception are described as: (1) increased K^+^ conductance and hyperpolarization of intrinsic neurons in the dorsal horn, (2) inhibition of the release of peptides, such as substance P and CGRP, and (3) inhibition of glutamate release [25,39,100,112,113]. According to [97], A_1_AR mechanisms also cause interneurons in the dorsal horn to release less γ-aminobutyric acid (GABA); however, the overall effect of this process is likely to be stimulatory, making it difficult to determine how much this action contributes to the suppression of pain.

It has been hypothesized that peripheral A_1_ARs physically associate with μ-opioid and α_2_-adrenergic receptors and generate tolerance, cross-tolerance, and cross-dependence with other drugs, i.e., heterologous desensitization, when an A_1_AR agonist is administered repeatedly [106]. The effectiveness of adenosine in such situations may be influenced by the activation of the α_2_-adrenergic receptor. Since those A_1_ARs have pronounced inhibitory activities, it is unclear what mechanism underlies this fundamentally excitatory function. This activity, meanwhile, highlights the possible complexity of A_1_AR responses to nerve damage, where a variety of spinal alterations in nociceptive transmission take place (e.g., central sensitization, disinhibition, phenotype switch).

Accordingly, using peripheral microdialysis, it was found that capsaicin (which activates TRPV1 receptors specifically expressed on C-fibers [114]), formalin (which causes neurogenic and tissue inflammation [115]), and glutamate [116] increased the peripheral extracellular levels of tissue adenosine. In each case, these actions were inhibited by capsaicin pretreatment, which indicated an interaction between A_1_ARs and TRPV1 receptors (see below).

Collectively, these findings suggest that directly acting agonists, as well as substances that boost the local tissue levels of extracellular adenosine, can activate inhibitory A_1_ARs on sensory afferents to suppress pain under specific circumstances (such as in the presence of a mild degree of stimulation, specific mediators, and nerve injury).

Unfortunately, despite their capacity to inhibit pain, the therapeutic advantage of A_1_AR agonists is constrained by their cardiovascular side effects [71]. Therefore, they are not currently being pursued for pain.

### 2.2. Adenosine A_2A_ and A_2B_ Receptors

The role of A_2A_AR and A_2B_AR in pain is more controversial, as their activation has both pro- and antinociceptive effects [45,52,117,118]. Furthermore, the peripheral activation of A_2A_AR and A_2B_AR, which have pronociceptive and vasodilatory properties, can result in the side effects of AR agonists. [44,49]. Regarding the potential mechanism of action of these receptors, A_2A_AR is coupled to Gs and Golf proteins, the latter mostly in the striatum [119]. Following the activation of these proteins, the stimulation of AC and increased cAMP synthesis are the highly significant intracellular events [15,49] that could stimulate cAMP-dependent kinase [120]. As a result, PKA can activate several pathways through the binding of PKC, influencing Ca^2+^ and K^+^ concentrations, cAMP-responsive elements [83], MAPK, and PLC activation [51,84,85,103]. Furthermore, the A_2B_AR receptor, which has a lesser affinity for adenosine compared to other ARs, promotes signaling by activating Gs and Gq. By stimulating PLC, A_2B_AR can increase IP3 and intracellular Ca^2+^ and stimulates the arachidonic acid pathway [49,121]. A_2B_AR may occasionally couple to Gq and Golf proteins as well [47]. It is known that both A_2A_ARs and A_2B_ARs, by increasing cAMP accumulation and activating downstream signal pathways, can lead to alterations in gene transcription relevant to the nociception effect. Increases in cAMP in sensory neurons cause PKA activation, Na^+^ channel phosphorylation, and activation of sensory afferents, all of which are mediators of hyperalgesia [81].

A_2A_ARs are highly expressed in the basal ganglia and the olfactory bulb of the CNS but are expressed at low levels in other brain areas, whereas A_2B_ARs and A_3_ARs are expressed at extremely low levels [73]. Moreover, the DRG sensory neurons contain adenosine A_2A_ARs [48] and A_2B_ARs [57]. The local peripheral injection of A_2A_AR agonists in the rat hind paw caused an increase in mechanical hyperalgesia in functional tests, as well as an increase in the flinching response to formalin [52].

A_2A_AR activation by extracellular adenosine resulted in an anti-inflammatory effect through its expression on diverse cell types [122]. In addition, the anti-inflammatory actions of the prototypical A_2A_AR agonist CGS21680 also included an increase in the serum interleukin (IL)-10 levels in complete Freund’s adjuvant (CFA)-injected rats, reflecting the data reported in lymphocytes from patients. In addition, treatment with CGS21680 induced a reduction in the secretion of proinflammatory cytokines in a murine calvary model of bone resorption induced by abrasion particles, while the IL-10 levels in the bone was considerably improved [54]. The local administration of CGS21680 in the hind paw led to mechanical hyperalgesia in rats [53], and the systemic administration of the A_2A_AR antagonist SCH 58261 attenuated the nociceptive responses in both acute and inflammatory tests in mice [27,63], which signifies a related role of A_2A_ARs in peripheral nociceptive signaling pathways. Previous studies demonstrated that LASSBio-1359, a novel A_2A_AR agonist, reversed the hyperalgesic response in mice, caused by the stimulation of the inflammatory process, through peripheral A_2A_AR activation [55].

Taiwo and Levine (1990) showed distinctly different effects of A_1_AR and A_2A_AR activation on peripheral pain [44]. They found that A_1_AR mediated analgesia in peripheral locations, while A_2A_AR enabled painful perception, as reported in other investigations [44,52,56,123,124]. However, the role of A_2A_AR is not clear. Indeed, other researchers showed a decrease in pain when A_2A_AR is activated [125,126,127]. These controversial results could be connected to the intracellular signaling of A_2A_AR. Its activation increases cAMP production, which could cause pain, but also opens the K^+^ channels, which could prevent pain [17,49,50].

Furthermore, A_2A_AR expressed on glial cells increased inflammatory mediator secretion, which caused and sustained chronic pain [90]. Therefore, A_2A_AR blocking may be a novel strategy for the treatment of neuropathic and chronic pain. A_2A_AR knockout animals are less sensitive to pain, suggesting that A_2A_AR antagonism is a pain reliever for acute [56] and chronic pain. The A_2A_AR agonist CGS21680, which was administered spinally [26,30] and supraspinally [50], was reported to produce antinociception. However, it was less active than A_1_AR agonists, and lacking the effects of selective A_2A_AR antagonists, it is unclear whether this receptor was responsible for such actions. Moreover, the A_2A_AR effects on spinal electrophysiology are complicated [100,112], and it is unclear which receptors cause these effects. The local administration of CGS21680 heightened the pain response [45,52], an effect that was likely mediated by the Gs-AC-PKA pathway. Intriguingly, one study found that forskolin and dibutyryl cAMP, which are PKA activators, increased the sensitivity to heat [128].

Contrarily, the activation of the adenosine receptor A_2A_AR increased nociception by sensitizing peripheral afferent axons that transmit to the spinal cord [56]. As a result, mice lacking the A_2A_AR responded less strongly to nociceptive thermal stimuli [117,118], whereas animals lacking the A_1_AR responded more strongly to nociceptive thermal stimuli [12,117]. Other research found no change in heat thresholds or antinociceptive reactions upon the systemic administration of morphine, a µ-opioid receptor agonist [129]. It was suggested that these functional variations in opioid actions reflected altered opioid receptor patterns in the spinal cord and, maybe, altered A_2A_ARs in sensory neurons. Because of the involvement of A_2A_ARs in inflammation [130] and the growing understanding of how inflammation contributes to neuropathic pain, responses to inflammation or nerve damage have not yet been described in A_2A_AR-null mice [131]. It has been shown that mice lacking A_2A_AR are hypoalgesic, which may indicate a peripheral pronociceptive role. [117].

The majority of inflammatory cells express A_2B_AR, and the activation of this receptor has both pro- and anti-inflammatory effects [58]. A_2B_AR has pro-inflammatory effects by promoting the secretion by mast cells and macrophages of IL-1, IL-13, IL-3, IL-8, IL-4, and VEGF [61]. Moreover, A_2B_AR induced the generation of IL-19 and TNF-α from bronchial epithelial cells [62]. The inhibition or deletion of the A_2B_ARs in mice reduced intestinal inflammation and slowed the progression of murine colitis/inflammatory bowel disease [132]. A_2B_ARs could be activated in pathological circumstances like hypoxia/ischemia and inflammation, where adenosine is considerably raised, as a larger quantity of adenosine is required for A_2B_AR activation compared to other AR subtypes [47,133]. A_2B_ARs are expressed both centrally and peripherally, in a wide variety of tissues, most notably in immune system cells. It has been suggested that these receptors, which have the lowest adenosine affinity compared to the other AR subtype, are particularly important in immunological processes like inflammation that are characterized by high adenosine concentrations. Several studies showed the peripheral pronociceptive impact of A_2B_AR activation [64,134], while the injection of A_2B_AR antagonists showed an analgesic effect. Other authors found that a prolonged enhancement in plasma adenosine activated A_2B_ARs on myeloid cells, resulting in the increase in circulating IL-6 and sIL-6R. Data on the advantages of using A_2B_AR antagonists in inflammatory processes, due to their anti-inflammatory actions [58,65] in addition to their analgesic effects [64], are available in the literature. However, there are limited data available on PSB-603, which is commercially available as a highly powerful and selective A_2B_AR antagonist in multiple species, including humans and rodents [135,136,137]. The tests carried out demonstrated that PSB-603 (i.p. 5 mg/kg) significantly decreased inflammation both in local and in systemic inflammation models. In particular, treatment with PSB-603 significantly decreased the quantities of both TNF-α and IL-6 and the levels of ROS in the paws of mice with inflammation generated according to the local inflammation model. The A_2B_AR antagonist did not affect the plasma levels of TNF-α and IL-6 in the systemic inflammation model. However, A_2B_AR activation was demonstrated to be excitotoxic even with heightened adenosine concentrations and would consequently initiate an inflammatory process [138]. The A_2B_AR antagonist was able to enhance the analgesic effects of morphine and paracetamol while decreasing thermal hyperalgesia [63,64]. A variety of A_2B_AR antagonists were also demonstrated to elicit antinociception when administered systemically; this effect was considered peripheral, since it was also seen in a structure that did not penetrate the central nervous system [64]. In the mouse tail immersion and hot plate tests, blocking A_2B_ARs enhanced the effects of paracetamol, and blocking A_2A_ARs had an antinociceptive effect even in the absence of paracetamol [63].

Mast cell mediators such as histamine and 5-hydroxytryptamine are released more often when A_2B_AR and A_3_AR are activated, which might augment pain signaling [17,24]. A_2A_AR agonists improved plasma extravasation when injected into the knee joint, although a local treatment with A_2A_AR, A_2B_AR, and A_3_AR agonists also caused edema that included mast cell degranulation [139,140,141]. These effects on the skin and joints are thought to be the result of pro-inflammatory activity.

Different methods revealed other factors that affect nociception. Although such action cannot be explained by A_1_AR inhibition, the analgesic properties of A_2A_AR and A_2B_AR antagonists have attracted interest.

### 2.3. Adenosine A_3_ Receptors

The exploration of the role of A_3_AR in pain and of the mechanisms and sites of action of A_3_AR agonists has begun recently. In many pain models, A_3_AR exhibited preclinical antinociceptive efficacy [5,6,7,71,142] and efficacy and safety in studies on non-painful states [7,143]. A_3_AR, like A_1_AR, is coupled to G_i/o_ proteins. Its coupling to the G_q/11_ protein is less well established. The inhibition of AC and the activation of PLC, IP3, DAG, PKC, and PLD, are the primary mechanisms involved in A_3_AR signaling. These receptors activate the MAPK pathway, namely, ERK1/2, as other ARs [67,68,72,143]. It was demonstrated that A_3_AR agonists reduced the pro-nociceptive N-type Ca^2+^ channels in rat DRG neurons [67,68] and by this mechanism inhibited post-visceral hypersensitivity [72]. More recent research showed that activating A_3_AR had an antihyperalgesic impact in many mouse models of neuropathic pain, which is in contrast with previous reports. Numerous preclinical pain models showed that A_3_AR ligands have significant anti-nociceptive effects [49,125]. Previous studies showed that the anti-nociceptive effects of endogenous adenosine depend on A_3_AR activation at spinal and supraspinal locations, suggesting that a selective A_3_AR agonist might be an effective analgesic [73]. The highly selective A_3_AR agonist MRS5698 decreased mechanical allodynia in numerous rat neuropathic pain models, including models of chronic constriction injury (CCI) and chemotherapy-induced neuropathic pain [49]. Previous research showed that the antinociceptive effects mediated by endogenous adenosine depend on A_3_AR activation at spinal and supraspinal locations, and pharmacological data identified a selective A_3_AR agonist as a potent non-narcotic drug [6].

Many cells are involved in the dual impact of A_3_AR on inflammatory processes, and this receptor subtype can have overlapping and opposing functions [64,144]. A_3_AR activation can also produce a pro-inflammatory effect by inducing the release of histamines and other allergic mediators [145], preventing eosinophilic chemotaxis and inhibiting apoptosis [69]. A_3_AR also boosted the fast infiltration of inflammatory cells by attracting eosinophils and macrophages in the lungs [146] and promoting Ca^2+^ signaling [147]. Concerning its role in pain, several previous publications described a pronociceptive profile of A_3_AR, since animals with its gene deletion demonstrated less hyperalgesia with inflammation and hypoalgesia in a nociceptive threshold test compared to control mice [14], but its effect in reducing chronic pain was revealed in subsequent studies.

The A_3_AR agonist IB-MECA was effective in increasing the mechanical threshold in a chronic inflammation model. However, the adenosine A_3_AR seems to have intricate effects on the CNS, with pro-inflammatory and anti-inflammatory functions, notably in healthy conditions. In agreement with this, previous studies indicated that IB-MECA reduced the IL-1 and IL-10 levels in the control group. Although A_3_AR is not highly expressed in the nervous system, adenosine has anti-inflammatory properties that can help relieve pain associated with peripheral inflammation [70]. A_3_AR agonists, such as IB-MECA, were previously tested in phase II and phase III clinical research for disorders other than pain and, so far, have demonstrated good safety profiles, in contrast to A_1_AR agonists, which have limited therapeutic application [148]. According to hot plate test results, mice lacking A_3_AR showed diminished nociception [149]. This was most likely due to a decline in the supraspinal processing and in the ability to “detect” painful stimuli. This is in line with the expression of A_3_AR in thalamic nuclei [150], where it may be involved in the processing of nociceptive inputs.

It is important to note that many of the earlier studies with supposedly A_1_AR-selective agonists, like R-PIA, have to be reinterpreted in light of the evidence that most of these agonists can also active A_3_AR in mouse models [151,152]. In addition to species differences in AR ligand affinity and efficacy, there are also species differences regarding the role of the receptor in inflammatory processes.

## 3. Allosteric Modulators

Using natural adenosine concentrations to activate the receptor in conjunction with an allosteric enhancer of the A_1_AR is an alternative method of promoting A_1_AR agonism. As this method depends on adenosine being created at the target area, it should have few side effects. Adenosine release is a normal compensatory process that occurs in some illness situations to assist tissues in regaining equilibrium.

In a rat model of plantar surgical damage, T-62, an A_1_AR positive allosteric modulator (PAM), reduced pain hypersensitivity in a dose-dependent manner (0.3–1 mcg i.t.). When clonidine was also provided, the amount of T-62 needed to produce an antihyperalgesic effect was cut in half, achieving only 40% of the maximum effect. Clinical testing of T-62 was conducted on patients with postherpetic neuralgia who were in pain [153,154,155]. Unfortunately, a clinical trial of the lead drug T-62 in post-herpetic neuropathic pain was discontinued [156]. However, future clinical trials of other A_1_AR allosteric enhancers might still be worthwhile.

The past 30 years witnessed a significant amount of research in the area of A_1_AR, which led to the development of clinical candidates for A_1_AR agonism, antagonism, and allosteric alteration. Pharmacologically speaking, the development of A_1_AR antagonists should be simpler than that of A_1_AR agonists because of the complications of developing A_1_AR agonists, such as receptor desensitization and the potential for severe cardiovascular and CNS side effects. The A_1_AR allosteric enhancer T-62 was previously evaluated in the clinic after showing encouraging effects in neuropathic pain animal models. Therapeutics that target A_1_AR (A_1_AR antagonists, A_1_AR agonists, and allosteric enhancers) may soon achieve long-awaited clinical success as a result of these significant scientific and clinical advancements. In addition to directly acting agonists and antagonists of A_1_AR and A_3_AR, allosteric modulators of these receptors have also been developed [69]. Allosteric modulators have the advantage of increasing the effects of endogenous adenosine released in response to stress at a specific location or tissue, causing a biological impact in the absence of an agonist. Numerous allosteric enhancers of the activation of ARs by agonists are being studied as therapeutic prospects.

The first known AR allosteric modulators were 2-aminothiophenes, exemplified by PD-81,723. Clinical trials are now examining the A_1_AR allosteric enhancer T-62 as a potential therapy for neuropathic pain. The difficulties in creating A_1_AR agonists, antagonists, or allosteric enhancers for therapeutic intervention in humans have now been well defined.

TRR469 was recently found to be one of the most potent A_1_AR positive allosteric modulators ever reported, with the ability to induce a 33-fold increase in adenosine affinity [155,157,158]. In the formalin and writhing tests, TRR469 successfully reduced nociceptive responses with results comparable to morphine. Additionally, unlike the orthosteric CCPA, it did not cause locomotion or cataleptic side effects in the diabetic neuropathic pain paradigm generated by streptozotocin (STZ) [158].

## 4. Clinical Studies

In several situations, it has been reported that the systemic administration of adenosine through i.v. infusion in humans (50–70 g/kg per minute over 45–60 min) resulted in some degree of pain relief. Adenosine infusions (i.v.) produced analgesia in healthy volunteers when used to treat experimental pain caused by cutaneous heat thresholds [159], ischemic pain [160,161], allodynia brought on by mustard oil [162], and cutaneous inflammatory pain [163]. In clinical studies including peripheral neuropathic pain [164,165] and mixed-etiology neuropathic pain, with postsurgical/posttraumatic neuropathic pain as the most common diagnosis [166], i.v. adenosine decreased spontaneous and evoked pain. In the latter experiment, which used an enhanced enrollment strategy, 40/66 patients (61%) were found to respond to adenosine during the initial open phase of the trial. In situations of neuropathic pain, one infusion of adenosine can occasionally result in long-term pain relief lasting months (estimated at 5–10% in [167], reported in 2/26 subjects in [165], and observed in 3/62 subjects in [166]). It is important to note that a comparable long-lasting pain relief from postherpetic neuralgia has been observed following a single [168] or repeated infusions of ATP, which is rapidly converted to adenosine [169]. When adenosine is administered during surgery, it also has analgesic effects on the patient. Segerdahl and colleagues found that adenosine (i.v.; 80 g/kg per minute) during surgery might decrease the need for anesthesia and/or postoperative analgesics [170,171,172]. Both medications stabilized the cardiovascular system during surgery, according to studies comparing intravenous adenosine infusions to intravenous remifentanil (a short-acting opioid) infusions [173,174]. However, the use of adenosine reduced postoperative pain and the need for opioid analgesics. The most striking effects (degree, duration of 48 h) and the lowest incidence of postoperative nausea were recorded by Fukunaga et al. [174]. They utilized a dose of 82 g/kg per minute of adenosine, with a cumulative dose of 2.5 g, compared to the dose of 17 g/kg per minute used by Zárate et al. [173], with a mean total dose of 1.4 mg.

It has been interesting to examine the possible effectiveness of adenosine administered spinally in humans in light of preclinical studies showing adenosine (i.t.) and A_1_AR agonists elicit antinociception [175]. Adenosine was shown to lessen experimentally produced allodynia from mustard oil [176] as well as hyperalgesia and allodynia after intradermal capsaicin injection in human volunteers when given intravenously (i.v) [177,178]. Adenosine (i.t.) decreased the area of allodynia 2–24 h post injection in a double-blind trial on individuals with neuropathic pain, supporting the observations of prior case reports [27,179]. Contrarily, adenosine (i.t.) had no impact on chronic pain following hysterectomy or on acute pain following the application of noxious heat [176,177]. According to a recent study, adenosine (i.t.) was unlikely to be helpful as an analgesic monotherapy due to its low efficacy (25% decrease in allodynia, no effect on continuous pain), the induction of back pain, and its inability to treat other types of pain [180].

Humans who received a peripheral adenosine injection experienced pain reactions similar to those brought on by ischemia circumstances [17]. Substance P [181] and nicotine [182] can increase the pain-initiating effects of adenosine, which are typically a limiting factor in the use of adenosine-related drugs for the treatment of chronic pain. Histamine and 5-hydroxytryptamine are released from mast cells upon activation of A_3_ARs, and these chemicals operate on the sensory nerve terminals, causing pain [17].

Peripheral adenosine injection has an inhibitory overall effect on pain processing, because increasing extracellular adenosine concentrations has an antinociceptive effect. Since adenosine has dual A_1_AR- and A_2A_AR-mediated anti-inflammatory and antinociceptive actions, there is growing interest in developing medicines that may have analgesic effects by altering the extracellular adenosine concentrations. Inhibitors of AK, whose spinally mediated antinociceptive effects were recognized more than ten years ago [23], are examples of effective medications. Orally administered AK inhibitors are more efficient at reducing inflammatory pain than neuropathic or acute pain, most likely because of the anti-inflammatory properties of adenosine [92]. According to studies comparing the antinociceptive and anti-inflammatory properties of AK inhibitors administered at the ipsilateral or contralateral sides of the injury, the antinociceptive action of AK inhibitors is systemically mediated at the level of the spinal dorsal horn [134]. The peripheral injection of an inflammatory agent causes enhanced c-Fos expression in the spinal dorsal horn, which could be decreased by AK inhibitors [134].

Antidepressants are frequently used to treat neuropathic pain; however, their analgesic effectiveness appears to occur without regard to any impact on one’s mood and may even include a rise in extracellular adenosine concentrations. This was demonstrated in rat models of neuropathic pain following either acute [183] or chronic [183] amitriptyline administration. In a rat model of excruciating diabetic neuropathy, endogenous adenosine also appeared to play a role in the antiallodynic activity of amitriptyline [184]. Esser and Sawynok (2000) [183] noted that amitriptyline reduced pain by modulating endogenous adenosine levels.

The analgesic effects of opioids may be influenced by rises in adenosine concentrations. Humans receiving i.t. morphine showed an increase in adenosine concentrations in their cerebrospinal fluid [179]. It is interesting to note that morphine-induced adenosine release is diminished in neuropathic rats [185], which may help to explain why opioids are less effective and potent at treating neuropathic pain. A_2A_AR-deficient animals were also found to have changes in the expression of various opioid receptor types, suggesting a functional interaction between A_2A_AR and opioid receptors in the modulation of pain [129].

A critical analysis of the uses of adenosine and ATP in pain control, which summarizes the majority of human investigations, indicated that adenosine compounds have a high potential to reduce pain [168]. This study emphasized that it is essential to consider medication dosages, routes, timing, and tissue penetration and that additional basic research is required to fully understand several issues. Caffeine can modulate pain through its antagonistic effects on ARs, but as recently highlighted [186], the type of effect (such as headache creation or relief) relies on the site of action, the dosage, and the timing of exposure. The interaction between paracetamol and coffee in pain control likely involves both A_2A_ARs and A_2B_ARs. Moreover, theophylline reduces chest discomfort in people with a hypersensitive esophageal mucosa, perhaps through modifying adenosine-mediated nociception [187]. Humans who received peripheral adenosine injection experienced pain reactions similar to those brought on by ischemia circumstances [17]. 

The use of A_1_AR agonists could be another strategy for treating migraine headaches by blocking the trigeminovascular system and CGRP. By acting on the trigeminal nucleus and preventing the release of CGRP into the bloodstream, A_1_AR activation limits trigeminovascular activation. This second effect is due to A_1_AR activation on peripheral trigeminal nerve terminals [16]. It was demonstrated that tonic A_1_AR activation prevents the facilitatory effects of CGRP in the hippocampus [188]. Interestingly, CGRP actions are promoted by A_2A_AR activation [188,189]; however, it is unclear how this information relates to using A_2A_AR antagonists as a migraine therapy strategy.

## 5. Conclusions

Adenosine receptors have been suggested as a possible target for the development of new analgesics due to the widespread capacity of A_1_AR agonists to attenuate pain responses in a variety of pain models after systemic administration. Nevertheless, in addition to producing antinociception, A_1_AR agonists also caused hypothermia, a reduction in locomotor activity, and cardiovascular alterations, and these effects are elicited by doses similar to those inducing antinociception. Non-nucleoside AK inhibitors are more effective in promoting antinociception, which has encouraged the development of indirectly acting drugs rather than directly acting agonists in the setting of a systemic administration [94]. Moreover, in several preclinical models of chronic pain, the regulation of A_3_AR results in strong anti-hypersensitive effects [70]. It is interesting to note that authors discovered that the A_1_AR-mediated antiallodynic effect was much shorter-lived than the A_2A_AR- or A_2B_AR-mediated pain control (lasting up to 6 weeks) [66]. As discussed above, these unorthodox pharmacological approaches have shown promising results in preclinical animal models of pain and could offer a solution to the challenges AR ligands have previously encountered in the clinic. Overall, the results reported in this review highlight the therapeutic potential of ARs as drug targets for the treatment of acute and chronic pain, as well as the need for the creation of novel, more effective approaches.

## Figures and Tables

**Table 1:** Expression and functional role of adenosine receptors in pain.

	Localization	Cellular Mechanisms	Preclinical Studies
**A_1_AR**	DRG, trigeminal ganglion spinal and supraspinal sites, laminae I and II of the dorsal horn, and descending projection within the posterior horn [17,35,36,37,38,39].	Trigger intracellular NO/cGMP/PKG/K_ATP_ signaling pathway [35]; Increase noradrenalin release [40,41]; Prevent neuronal activity in the spinal cord and DRGs [25,42,43].	***Reduce*:** Formalin-induced pain and hyperalgesia induced by PGE2 [44,45]; Thermal and mechanical hyperalgesia induced by spinal nerve ligation in rats [15,16,45] Arthritis-induced pain and neuropathy-induced pain [46]
**A_2A_AR**	DRG, basal ganglia, and the olfactory bulb of the CNS [47,48].	Increase cAMP production and open K^+^ channels [17,49,50]; Increase inflammatory mediators released by glial cells [51].	***Increase*:** Mechanical hyperalgesia and flinching response to formalin [52]; Mechanical hyperalgesia in rats [53]. ***Reduce*:** Secretion of proinflammatory cytokines [54]; Hyperalgesic response induced by activation of the peripheral receptor in mice [55]. A_2A_AR knockout animals are less sensitive to pain [56].
**A_2B_AR**	DRG, spinal cord, astrocyte, and inflammatory cells [57,58,59,60].	Induce secretion of IL-6 from macrophages and of IL-1, IL-13, IL-3, IL-8, IL-4, and VEGF from mast cells [61]; Induce the generation of IL-19 and TNF-α from bronchial epithelial cells [62].	***Block of A_2B_ARs*:** Decreases thermal hyperalgesia [63,64]; Analgesic impact on inflammatory pain [65].
**A_3_AR**	DRG, spinal and supraspinal locations [66,67,68].	Reduce pro-nociceptive N-type Ca^2+^ channels in rat DRG [68]; Induce release of histamines [59]; Prevent eosinophilic chemotaxis and inhibit apoptosis [69]; Increase anti-inflammatory mediators release [70].	***Reduce*:** Neuropathic pain induced by CCI or chemotherapy treatment in rodents [4,66]; Mechanical allodynia in various rat neuropathic pain models [71]; Acute visceral pain [72].

Abbreviations: dorsal root ganglia (DRG); nitric oxide (NO); cyclic guanosine monophosphate (cGMP); protein kinase G (PKG); ATP-sensitive channels (K_ATP_); prostaglandin E2 (PGE2); central nervous system (CNS); cyclic AMP (cAMP); interleukin (IL); vascular endothelial growth factor (VEGF); tumor necrosis factor-alpha (TNF-α); chronic constriction injury of the sciatic nerve (CCI).

## Data Availability

Not applicable.

## References

[1] Luongo L., Guida F., Maione S., Jacobson K.A., Salvemini D. (2021). Adenosine Metabotropic Receptors in Chronic Pain Management. Front. Pharmacol..

[2] Vincenzi F., Pasquini S., Borea P.A., Varani K. (2020). Targeting Adenosine Receptors: A Potential Pharmacological Avenue for Acute and Chronic Pain. Int. J. Mol. Sci..

[3] Nahin R.L., Feinberg T., Kapos F.P., Terman G.W. (2023). Estimated Rates of Incident and Persistent Chronic Pain among US Adults, 2019–2020. JAMA Netw. Open.

[4] Chen Z., Janes K., Chen C., Doyle T., Bryant L., Tosh D.K., Jacobson K.A., Salvemini D. (2012). Controlling murine and rat chronic pain through A_3_ adenosine receptor activation. FASEB J..

[5] Ford A., Castonguay A., Cottet M., Little J.W., Chen Z., Symons-Liguori A.M., Doyle T., Egan T.M., Vanderah T.W., De Koninck Y. (2015). Engagement of the GABA to KCC2 signaling pathway contributes to the analgesic effects of A3AR agonists in neuropathic pain. J. Neurosci..

[6] Little J.W., Ford A., Symons-Liguori A.M., Chen Z., Janes K., Doyle T., Xie J., Luongo L., Tosh D.K., Maione S. (2015). Endogenous adenosine A_3_ receptor activation selectively alleviates persistent pain states. Brain.

[7] Janes K., Symons-Liguori A.M., Jacobson K.A., Salvemini D. (2016). Identification of A_3_ adenosine receptor agonists as novel non-narcotic analgesics. Br. J. Pharmacol..

[8] Jacobson K.A., Giancotti L.A., Lauro F., Mufti F., Salvemini D. (2020). Treatment of chronic neuropathic pain: Purine receptor modulation. Pain.

[9] Haddad M., Alsalem M., Saleh T., Jaffal S.M., Barakat N.A., El-Salem K. (2023). Interaction of the synthetic cannabinoid WIN55212 with tramadol on nociceptive thresholds and core body temperature in a chemotherapy-induced peripheral neuropathy pain model. Neuroreport.

[10] Haddad M. (2021). The Impact of CB1 Receptor on Inflammation in Skeletal Muscle Cells. J. Inflamm. Res..

[11] Kotańska M., Szafarz M., Mika K., Dziubina A., Bednarski M., Müller C.E., Sapa J., Kieć-Kononowicz K. (2021). PSB 603—A known selective adenosine A_2_B receptor antagonist—Has anti-inflammatory activity in mice. Biomed. Pharmacother..

[12] Wu W.P., Hao J.X., Halldner L., Lövdahl C., DeLander G.E., Wiesenfeld-Hallin Z., Fredholm B.B., Xu X.J. (2005). Increased nociceptive response in mice lacking the adenosine A_1_ receptor. Pain.

[13] Haddad M., Alsalem M., Aldossary S.A., Kalbouneh H., Jaffal S.M., Alshawabkeh Q., Al Hayek S., Abdelhai O., Barakat N.A., El-Salem K. (2023). The role of adenosine receptor ligands on inflammatory pain: Possible modulation of TRPV1 receptor function. Inflammopharmacology.

[14] Wu W.P., Hao J.X., Halldner-Henriksson L., Xu X.J., Jacobson M.A., Wiesenfeld-Hallin Z., Fredholm B.B. (2002). Decreased inflammatory pain due to reduced carrageenan-induced inflammation in mice lacking adenosine A_3_ receptors. Neuroscience.

[15] Sawynok J., Liu X.J. (2003). Adenosine in the spinal cord and periphery: Release and regulation of pain. Prog. Neurobiol..

[16] Goadsby P.J., Hoskin K.L., Storer R.J., Edvinsson L., Connor H.E. (2002). Adenosine A_1_ receptor agonists inhibit trigeminovascular nociceptive transmission. Brain.

[17] Sawynok J. (1998). Adenosine receptor activation and nociception. Eur. J. Pharmacol..

[18] Antonioli L., Colucci R., La Motta C., Tuccori M., Awwad O., Da Settimo F., Blandizzi C., Fornai M. (2012). Adenosine deaminase in the modulation of immune system and its potential as a novel target for treatment of inflammatory disorders. Curr. Drug Targets.

[19] Pasquini S., Contri C., Borea P.A., Vincenzi F., Varani K. (2021). Adenosine and Inflammation: Here, There and Everywhere. Int. J. Mol. Sci..

[20] Jacobson K.A., Reitman M.L. (2020). Adenosine-Related Mechanisms in Non-Adenosine Receptor Drugs. Cells.

[21] Gomes J.A., Li X., Pan H.L., Eisenach J.C. (1999). Intrathecal adenosine interacts with a spinal noradrenergic system to produce antinociception in nerve-injured rats. Anesthesiology.

[22] Karlsten R., Gordh T., Hartvig P., Post C. (1990). Effects of intrathecal injection of the adenosine receptor agonists R-phenylisopropyl-adenosine and N-ethylcarboxamide-adenosine on nociception and motor function in the rat. Anesth. Analg..

[23] Keil G.J., DeLander G.E. (1992). Spinally-mediated antinociception is induced in mice by an adenosine kinase-, but not by an adenosine deaminase-, inhibitor. Life Sci..

[24] Sawynok J., Reid A., Poon A. (1998). Peripheral antinociceptive effect of an adenosine kinase inhibitor, with augmentation by an adenosine deaminase inhibitor, in the rat formalin test. Pain.

[25] Liu X.J., Salter M.W. (2005). Purines and pain mechanisms: Recent developments. Curr. Opin. Investig. Drugs.

[26] Lee Y.W., Yaksh T.L. (1996). Pharmacology of the spinal adenosine receptor which mediates the antiallodynic action of intrathecal adenosine agonists. J. Pharmacol. Exp. Ther..

[27] Belfrage M., Segerdahl M., Arnér S., Sollevi A. (1999). The safety and efficacy of intrathecal adenosine in patients with chronic neuropathic pain. Anesth. Analg..

[28] Karlsten R., Gordh T. (1995). An A_1_-selective adenosine agonist abolishes allodynia elicited by vibration and touch after intrathecal injection. Anesth. Analg..

[29] Yang J., Hsieh C.L., Lin Y.W. (2017). Role of Transient Receptor Potential Vanilloid 1 in Electroacupuncture Analgesia on Chronic Inflammatory Pain in Mice. Biomed Res. Int..

[30] Poon A., Sawynok J. (1998). Antinociception by adenosine analogs and inhibitors of adenosine metabolism in an inflammatory thermal hyperalgesia model in the rat. Pain.

[31] Kowaluk E.A., Jarvis M.F. (2000). Therapeutic potential of adenosine kinase inhibitors. Expert Opin. Investig. Drugs.

[32] Sosnowski M., Stevens C.W., Yaksh T.L. (1989). Assessment of the role of A_1_/A_2_ adenosine receptors mediating the purine antinociception, motor and autonomic function in the rat spinal cord. J. Pharmacol. Exp. Ther..

[33] Lavand’homme P.M., Eisenach J.C. (1999). Exogenous and endogenous adenosine enhance the spinal antiallodynic effects of morphine in a rat model of neuropathic pain. Pain.

[34] Cronstein B.N., Levin R.I., Philips M., Hirschhorn R., Abramson S.B., Weissmann G. (1992). Neutrophil adherence to endothelium is enhanced via adenosine A_1_ receptors and inhibited via adenosine A_2_ receptors. J. Immunol..

[35] Lima F.O., Souza G.R., Verri W.A., Parada C.A., Ferreira S.H., Cunha F.Q., Cunha T.M. (2010). Direct blockade of inflammatory hypernociception by peripheral A_1_ adenosine receptors: Involvement of the NO/cGMP/PKG/KATP signaling pathway. Pain.

[36] Metzner K., Gross T., Balzulat A., Wack G., Lu R., Schmidtko A. (2021). Lack of efficacy of a partial adenosine A_1_ receptor agonist in neuropathic pain models in mice. Purinergic Signal..

[37] Schulte G., Robertson B., Fredholm B.B., DeLander G.E., Shortland P., Molander C. (2003). Distribution of antinociceptive adenosine A_1_ receptors in the spinal cord dorsal horn, and relationship to primary afferents and neuronal subpopulations. Neuroscience.

[38] Choca J.I., Green R.D., Proudfit H.K. (1988). Adenosine A1 and A2 receptors of the substantia gelatinosa are located predominantly on intrinsic neurons: An autoradiography study. J. Pharmacol. Exp. Ther..

[39] Ackley M.A., Governo R.J., Cass C.E., Young J.D., Baldwin S.A., King A.E. (2003). Control of glutamatergic neurotransmission in the rat spinal dorsal horn by the nucleoside transporter ENT1. J. Physiol..

[40] Bantel C., Tobin J.R., Li X., Childers S.R., Chen S.R., Eisenach J.C. (2002). Intrathecal adenosine following spinal nerve ligation in rat: Short residence time in cerebrospinal fluid and no change in A(1) receptor binding. Anesthesiology.

[41] Bantel C., Childers S.R., Eisenach J.C. (2002). Role of adenosine receptors in spinal G-protein activation after peripheral nerve injury. Anesthesiology.

[42] Deuchars S.A., Brooke R.E., Deuchars J. (2001). Adenosine A_1_ receptors reduce release from excitatory but not inhibitory synaptic inputs onto lateral horn neurons. J. Neurosci..

[43] Reeve A.J., Dickenson A.H. (1995). Electrophysiological study on spinal antinociceptive interactions between adenosine and morphine in the dorsal horn of the rat. Neurosci. Lett..

[44] Taiwo Y.O., Levine J.D. (1990). Direct cutaneous hyperalgesia induced by adenosine. Neuroscience.

[45] Karlsten R., Gordh T., Post C. (1992). Local antinociceptive and hyperalgesic effects in the formalin test after peripheral administration of adenosine analogues in mice. Pharmacol. Toxicol..

[46] Curros-Criado M.M., Herrero J.F. (2005). The antinociceptive effects of the systemic adenosine A_1_ receptor agonist CPA in the absence and in the presence of spinal cord sensitization. Pharmacol. Biochem. Behav..

[47] Fredholm B.B., IJzerman A.P., Jacobson K.A., Linden J., Müller C.E. (2011). International Union of Basic and Clinical Pharmacology. LXXXI. Nomenclature and classification of adenosine receptors—An update. Pharmacol. Rev..

[48] Kaelin-Lang A., Lauterburg T., Burgunder J.M. (1998). Expression of adenosine A2a receptor gene in rat dorsal root and autonomic ganglia. Neurosci. Lett..

[49] Jacobson K.A., Gao Z.G. (2006). Adenosine receptors as therapeutic targets. Nat. Rev. Drug Discov..

[50] Regaya I., Pham T., Andreotti N., Sauze N., Carrega L., Martin-Eauclaire M.F., Jouirou B., Peragut J.C., Vacher H., Rochat H. (2004). Small conductance calcium-activated K+ channels, SkCa, but not voltage-gated K+ (Kv) channels, are implicated in the antinociception induced by CGS21680, a A_2_A adenosine receptor agonist. Life Sci..

[51] Boison D., Chen J.F., Fredholm B.B. (2010). Adenosine signaling and function in glial cells. Cell Death Differ..

[52] Doak G.J., Sawynok J. (1995). Complex role of peripheral adenosine in the genesis of the response to subcutaneous formalin in the rat. Eur. J. Pharmacol..

[53] Khasar S.G., Wang J.F., Taiwo Y.O., Heller P.H., Green P.G., Levine J.D. (1995). Mu-opioid agonist enhancement of prostaglandin-induced hyperalgesia in the rat: A G-protein beta gamma subunit-mediated effect?. Neuroscience.

[54] Mediero A., Frenkel S.R., Wilder T., He W., Mazumder A., Cronstein B.N. (2012). Adenosine A_2_A receptor activation prevents wear particle-induced osteolysis. Sci. Transl. Med..

[55] Montes G.C., Hammes N., da Rocha M.D., Montagnoli T.L., Fraga C.A., Barreiro E.J., Sudo R.T., Zapata-Sudo G. (2016). Treatment with Adenosine Receptor Agonist Ameliorates Pain Induced by Acute and Chronic Inflammation. J. Pharmacol. Exp. Ther..

[56] Hussey M.J., Clarke G.D., Ledent C., Hourani S.M.O., Kitchen I. (2007). Reduced response to the formalin test and lowered spinal NMDA glutamate receptor binding in adenosine A_2_A receptor knockout mice. Pain.

[57] Li W., Dai D., Chen A., Gao X.F., Xiong L. (2022). Characteristics of Zusanli Dorsal Root Ganglion Neurons in Rats and Their Receptor Mechanisms in Response to Adenosine. J. Pain.

[58] Antonioli L., Csóka B., Fornai M., Colucci R., Kókai E., Blandizzi C., Haskó G. (2014). Adenosine and inflammation: What’s new on the horizon?. Drug Discov. Today.

[59] Borea P.A., Gessi S., Merighi S., Vincenzi F., Varani K. (2018). Pharmacology of Adenosine Receptors: The State of the Art. Physiol. Rev..

[60] Popoli P., Pepponi R. (2012). Potential therapeutic relevance of adenosine A_2_B and A_2_A receptors in the central nervous system. CNS Neurol. Disord. Drug Targets.

[61] Ryzhov S., Zaynagetdinov R., Goldstein A.E., Novitskiy S.V., Blackburn M.R., Biaggioni I., Feoktistov I. (2008). Effect of A2B adenosine receptor gene ablation on adenosine-dependent regulation of proinflammatory cytokines. J. Pharmacol. Exp. Ther..

[62] Zhong H., Wu Y., Belardinelli L., Zeng D. (2006). A2B adenosine receptors induce IL-19 from bronchial epithelial cells, resulting in TNF-alpha increase. Am. J. Respir. Cell Mol. Biol..

[63] Godfrey L., Yan L., Clarke G.D., Ledent C., Kitchen I., Hourani S.M. (2006). Modulation of paracetamol antinociception by caffeine and by selective adenosine A_2_ receptor antagonists in mice. Eur. J. Pharmacol..

[64] Abo-Salem O.M., Hayallah A.M., Bilkei-Gorzo A., Filipek B., Zimmer A., Müller C.E. (2004). Antinociceptive effects of novel A2B adenosine receptor antagonists. J. Pharmacol. Exp. Ther..

[65] Bilkei-Gorzo A., Abo-Salem O.M., Hayallah A.M., Michel K., Müller C.E., Zimmer A. (2008). Adenosine receptor subtype-selective antagonists in inflammation and hyperalgesia. Naunyn-Schmiedeberg’s Arch. Pharmacol..

[66] Coppi E., Cherchi F., Lucarini E., Ghelardini C., Pedata F., Jacobson K.A., Di Cesare Mannelli L., Pugliese A.M., Salvemini D. (2021). Uncovering the Mechanisms of Adenosine Receptor-Mediated Pain Control: Focus on the A. Int. J. Mol. Sci..

[67] Cherchi F., Venturini M., Magni G., Scortichini M., Jacobson K.A., Pugliese A.M., Coppi E. (2023). Covalently Binding Adenosine A. Purinergic Signal..

[68] Coppi E., Cherchi F., Fusco I., Failli P., Vona A., Dettori I., Gaviano L., Lucarini E., Jacobson K.A., Tosh D.K. (2019). Adenosine A_3_ receptor activation inhibits pronociceptive N-type Ca^2+^ currents and cell excitability in dorsal root ganglion neurons. Pain.

[69] Gao Z., Li B.S., Day Y.J., Linden J. (2001). A_3_ adenosine receptor activation triggers phosphorylation of protein kinase B and protects rat basophilic leukemia 2H3 mast cells from apoptosis. Mol. Pharmacol..

[70] Durante M., Squillace S., Lauro F., Giancotti L.A., Coppi E., Cherchi F., Di Cesare Mannelli L., Ghelardini C., Kolar G., Wahlman C. (2021). Adenosine A_3_ agonists reverse neuropathic pain via T cell-mediated production of IL-10. J. Clin. Investig..

[71] Janes K., Wahlman C., Little J.W., Doyle T., Tosh D.K., Jacobson K.A., Salvemini D. (2015). Spinal neuroimmune activation is independent of T-cell infiltration and attenuated by A3 adenosine receptor agonists in a model of oxaliplatin-induced peripheral neuropathy. Brain Behav. Immun..

[72] Lucarini E., Coppi E., Micheli L., Parisio C., Vona A., Cherchi F., Pugliese A.M., Pedata F., Failli P., Palomino S. (2020). Acute visceral pain relief mediated by A3AR agonists in rats: Involvement of N-type voltage-gated calcium channels. Pain.

[73] Fredholm B.B., IJzerman A.P., Jacobson K.A., Klotz K.N., Linden J. (2001). International Union of Pharmacology. XXV. Nomenclature and classification of adenosine receptors. Pharmacol. Rev..

[74] Antonioli L., Blandizzi C., Pacher P., Haskó G. (2019). The Purinergic System as a Pharmacological Target for the Treatment of Immune-Mediated Inflammatory Diseases. Pharmacol. Rev..

[75] Antonioli L., Pacher P., Vizi E.S., Haskó G. (2013). CD39 and CD73 in immunity and inflammation. Trends Mol. Med..

[76] Boison D. (2013). Adenosine kinase: Exploitation for therapeutic gain. Pharmacol. Rev..

[77] Baldwin S.A., Beal P.R., Yao S.Y., King A.E., Cass C.E., Young J.D. (2004). The equilibrative nucleoside transporter family, SLC29. Pflugers Arch..

[78] Pedata F., Corsi C., Melani A., Bordoni F., Latini S. (2001). Adenosine extracellular brain concentrations and role of A_2A_ receptors in ischemia. Ann. N. Y Acad. Sci..

[79] Dunwiddie T.V., Diao L. (1994). Extracellular adenosine concentrations in hippocampal brain slices and the tonic inhibitory modulation of evoked excitatory responses. J. Pharmacol. Exp. Ther..

[80] Fredholm B.B., Dunwiddie T.V., Bergman B., Lindström K. (1984). Levels of adenosine and adenine nucleotides in slices of rat hippocampus. Brain Res..

[81] Gold M.S., Reichling D.B., Shuster M.J., Levine J.D. (1996). Hyperalgesic agents increase a tetrodotoxin-resistant Na+ current in nociceptors. Proc. Natl. Acad. Sci. USA.

[82] Chen J.F., Lee C.F., Chern Y. (2014). Adenosine receptor neurobiology: Overview. Int. Rev. Neurobiol..

[83] Liou J.T., Liu F.C., Hsin S.T., Yang C.Y., Lui P.W. (2007). Inhibition of the cyclic adenosine monophosphate pathway attenuates neuropathic pain and reduces phosphorylation of cyclic adenosine monophosphate response element-binding in the spinal cord after partial sciatic nerve ligation in rats. Anesth. Analg..

[84] Burnstock G. (2007). Purine and pyrimidine receptors. Cell. Mol. Life Sci..

[85] Burnstock G. (2007). Physiology and pathophysiology of purinergic neurotransmission. Physiol. Rev..

[86] Chuang H.H., Prescott E.D., Kong H., Shields S., Jordt S.E., Basbaum A.I., Chao M.V., Julius D. (2001). Bradykinin and nerve growth factor release the capsaicin receptor from PtdIns(4,5)P2-mediated inhibition. Nature.

[87] Morales-Lázaro S.L., Simon S.A., Rosenbaum T. (2013). The role of endogenous molecules in modulating pain through transient receptor potential vanilloid 1 (TRPV1). J. Physiol..

[88] Fredholm B.B., Assender J.W., Irenius E., Kodama N., Saito N. (2003). Synergistic effects of adenosine A_1_ and P2Y receptor stimulation on calcium mobilization and PKC translocation in DDT1 MF-2 cells. Cell. Mol. Neurobiol..

[89] Fredholm B.B. (2003). Adenosine receptors as targets for drug development. Drug News Perspect..

[90] Keil G.J., DeLander G.E. (1994). Adenosine kinase and adenosine deaminase inhibition modulate spinal adenosine- and opioid agonist-induced antinociception in mice. Eur. J. Pharmacol..

[91] McGaraughty S., Cowart M., Jarvis M.F. (2001). Recent developments in the discovery of novel adenosine kinase inhibitors: Mechanism of action and therapeutic potential. CNS Drug Rev..

[92] Jarvis M.F., Burgard E.C., McGaraughty S., Honore P., Lynch K., Brennan T.J., Subieta A., Van Biesen T., Cartmell J., Bianchi B. (2002). A-317491, a novel potent and selective non-nucleotide antagonist of P2X3 and P2X2/3 receptors, reduces chronic inflammatory and neuropathic pain in the rat. Proc. Natl. Acad. Sci. USA.

[93] Zhu C.Z., Mikusa J., Chu K.L., Cowart M., Kowaluk E.A., Jarvis M.F., McGaraughty S. (2001). A-134974: A novel adenosine kinase inhibitor, relieves tactile allodynia via spinal sites of action in peripheral nerve injured rats. Brain Res..

[94] McGaraughty S., Cowart M., Jarvis M.F., Berman R.F. (2005). Anticonvulsant and antinociceptive actions of novel adenosine kinase inhibitors. Curr. Top. Med. Chem..

[95] Golembiowska K., White T.D., Sawynok J. (1996). Adenosine kinase inhibitors augment release of adenosine from spinal cord slices. Eur. J. Pharmacol..

[96] DeLander G.E., Wahl J.J. (1989). Morphine (intracerebroventricular) activates spinal systems to inhibit behavior induced by putative pain neurotransmitters. J. Pharmacol. Exp. Ther..

[97] Hugel S., Schlichter R. (2003). Convergent control of synaptic GABA release from rat dorsal horn neurones by adenosine and GABA autoreceptors. J. Physiol..

[98] Geiger J.D., Nagy J.I. (1984). Heterogeneous distribution of adenosine transport sites labelled by [3H]nitrobenzylthioinosine in rat brain: An autoradiographic and membrane binding study. Brain Res. Bull..

[99] Dolphin A.C., Forda S.R., Scott R.H. (1986). Calcium-dependent currents in cultured rat dorsal root ganglion neurones are inhibited by an adenosine analogue. J. Physiol..

[100] Lao L.J., Kumamoto E., Luo C., Furue H., Yoshimura M. (2001). Adenosine inhibits excitatory transmission to substantia gelatinosa neurons of the adult rat spinal cord through the activation of presynaptic Aadenosine receptor. Pain.

[101] DeLander G.E., Wahl J.J. (1988). Behavior induced by putative nociceptive neurotransmitters is inhibited by adenosine or adenosine analogs coadministered intrathecally. J. Pharmacol. Exp. Ther..

[102] Nakamura I., Ohta Y., Kemmotsu O. (1997). Characterization of adenosine receptors mediating spinal sensory transmission related to nociceptive information in the rat. Anesthesiology.

[103] Burnstock G. (2011). Introductory overview of purinergic signalling. Front. Biosci. -Elite.

[104] Song J.G., Hahm K.D., Kim Y.K., Leem J.G., Lee C., Jeong S.M., Park P.H., Shin J.W. (2011). Adenosine triphosphate-sensitive potassium channel blockers attenuate the antiallodynic effect of R-PIA in neuropathic rats. Anesth. Analg..

[105] Horiuchi H., Ogata T., Morino T., Yamamoto H. (2010). Adenosine A_1_ receptor agonists reduce hyperalgesia after spinal cord injury in rats. Spinal Cord..

[106] Carruthers A.M., Sellers L.A., Jenkins D.W., Jarvie E.M., Feniuk W., Humphrey P.P. (2001). Adenosine A_1_ receptor-mediated inhibition of protein kinase A-induced calcitonin gene-related peptide release from rat trigeminal neurons. Mol. Pharmacol..

[107] Haas H.L., Selbach O. (2000). Functions of neuronal adenosine receptors. Naunyn-Schmiedeberg’s Arch. Pharmacol..

[108] Nascimento F.P., Figueredo S.M., Marcon R., Martins D.F., Macedo S.J., Lima D.A., Almeida R.C., Ostroski R.M., Rodrigues A.L., Santos A.R. (2010). Inosine reduces pain-related behavior in mice: Involvement of adenosine A_1_ and A_2A_ receptor subtypes and protein kinase C pathways. J. Pharmacol. Exp. Ther..

[109] Zahn P.K., Straub H., Wenk M., Pogatzki-Zahn E.M. (2007). Adenosine A_1_ but not A_2a_ receptor agonist reduces hyperalgesia caused by a surgical incision in rats: A pertussis toxin-sensitive G protein-dependent process. Anesthesiology.

[110] Johansson B., Halldner L., Dunwiddie T.V., Masino S.A., Poelchen W., Giménez-Llort L., Escorihuela R.M., Fernández-Teruel A., Wiesenfeld-Hallin Z., Xu X.J. (2001). Hyperalgesia, anxiety, and decreased hypoxic neuroprotection in mice lacking the adenosine A_1_ receptor. Proc. Natl. Acad. Sci. USA.

[111] Taiwo Y.O., Levine J.D. (1991). Further confirmation of the role of adenyl cyclase and of cAMP-dependent protein kinase in primary afferent hyperalgesia. Neuroscience.

[112] Patel M.K., Pinnock R.D., Lee K. (2001). Adenosine exerts multiple effects in dorsal horn neurones of the adult rat spinal cord. Brain Res..

[113] Yamamoto S., Nakanishi O., Matsui T., Shinohara N., Kinoshita H., Lambert C., Ishikawa T. (2003). Intrathecal adenosine A_1_ receptor agonist attenuates hyperalgesia without inhibiting spinal glutamate release in the rat. Cell. Mol. Neurobiol..

[114] Liu X.J., White T.D., Sawynok J. (2001). Involvement of primary sensory afferents, postganglionic sympathetic nerves and mast cells in the formalin-evoked peripheral release of adenosine. Eur. J. Pharmacol..

[115] Liu X.J., White T.D., Sawynok J. (2000). Potentiation of formalin-evoked adenosine release by an adenosine kinase inhibitor and an adenosine deaminase inhibitor in the rat hind paw: A microdialysis study. Eur. J. Pharmacol..

[116] Liu X.J., White T.D., Sawynok J. (2002). Intraplantar injection of glutamate evokes peripheral adenosine release in the rat hind paw: Involvement of peripheral ionotropic glutamate receptors and capsaicin-sensitive sensory afferents. J. Neurochem..

[117] Ledent C., Vaugeois J.M., Schiffmann S.N., Pedrazzini T., El Yacoubi M., Vanderhaeghen J.J., Costentin J., Heath J.K., Vassart G., Parmentier M. (1997). Aggressiveness, hypoalgesia and high blood pressure in mice lacking the adenosine A_2a_ receptor. Nature.

[118] Berrendero F., Castañé A., Ledent C., Parmentier M., Maldonado R., Valverde O. (2003). Increase of morphine withdrawal in mice lacking A_2a_ receptors and no changes in C_B1_/A_2a_ double knockout mice. Eur. J. Neurosci..

[119] Xie K., Masuho I., Shih C.C., Cao Y., Sasaki K., Lai C.W., Han P.L., Ueda H., Dessauer C.W., Ehrlich M.E. (2015). Stable G protein-effector complexes in striatal neurons: Mechanism of assembly and role in neurotransmitter signaling. Elife.

[120] Parada C.A., Reichling D.B., Levine J.D. (2005). Chronic hyperalgesic priming in the rat involves a novel interaction between cAMP and PKCepsilon second messenger pathways. Pain.

[121] Feoktistov I., Biaggioni I. (2011). Role of adenosine A_2B_ receptors in inflammation. Adv. Pharmacol..

[122] Haskó G., Pacher P. (2008). A_2_A receptors in inflammation and injury: Lessons learned from transgenic animals. J. Leukoc. Biol..

[123] Bastia E., Varani K., Monopoli A., Bertorelli R. (2002). Effects of A_1_ and A_2A_ adenosine receptor ligands in mouse acute models of pain. Neurosci. Lett..

[124] Li L., Hao J.X., Fredholm B.B., Schulte G., Wiesenfeld-Hallin Z., Xu X.J. (2010). Peripheral adenosine A_2_A receptors are involved in carrageenan-induced mechanical hyperalgesia in mice. Neuroscience.

[125] Yoon M.H., Bae H.B., Choi J.I. (2005). Antinociception of intrathecal adenosine receptor subtype agonists in rat formalin test. Anesth. Analg..

[126] Borghi V., Przewlocka B., Labuz D., Maj M., Ilona O., Pavone F. (2002). Formalin-induced pain and mu-opioid receptor density in brain and spinal cord are modulated by A_1_ and A_2a_ adenosine agonists in mice. Brain Res..

[127] By Y., Condo J., Durand-Gorde J.M., Lejeune P.J., Mallet B., Guieu R., Ruf J. (2011). Intracerebroventricular injection of an agonist-like monoclonal antibody to adenosine A_2A_ receptor has antinociceptive effects in mice. J. Neuroimmunol..

[128] Mizumura K., Koda H., Kumazawa T. (1996). Opposite effects of increased intracellular cyclic AMP on the heat and bradykinin responses of canine visceral polymodal receptors in vitro. Neurosci. Res..

[129] Bailey A., Ledent C., Kelly M., Hourani S.M., Kitchen I. (2002). Changes in spinal delta and kappa opioid systems in mice deficient in the A_2A_ receptor gene. J. Neurosci..

[130] Ohta A., Sitkovsky M. (2001). Role of G-protein-coupled adenosine receptors in downregulation of inflammation and protection from tissue damage. Nature.

[131] Watkins L.R., Maier S.F. (2002). Beyond neurons: Evidence that immune and glial cells contribute to pathological pain states. Physiol. Rev..

[132] Antonioli L., Blandizzi C., Csóka B., Pacher P., Haskó G. (2015). Adenosine signalling in diabetes mellitus--pathophysiology and therapeutic considerations. Nat. Rev. Endocrinol..

[133] Borea P.A., Gessi S., Merighi S., Vincenzi F., Varani K. (2017). Pathological overproduction: The bad side of adenosine. Br. J. Pharmacol..

[134] Poon A., Sawynok J. (1999). Antinociceptive and anti-inflammatory properties of an adenosine kinase inhibitor and an adenosine deaminase inhibitor. Eur. J. Pharmacol..

[135] Schiedel A.C., Hinz S., Thimm D., Sherbiny F., Borrmann T., Maass A., Müller C.E. (2011). The four cysteine residues in the second extracellular loop of the human adenosine A_2B_ receptor: Role in ligand binding and receptor function. Biochem. Pharmacol..

[136] Alnouri M.W., Jepards S., Casari A., Schiedel A.C., Hinz S., Müller C.E. (2015). Selectivity is species-dependent: Characterization of standard agonists and antagonists at human, rat, and mouse adenosine receptors. Purinergic Signal..

[137] Shakya A.K., Naik R.R., Almasri I.M., Kaur A. (2019). Role and Function of Adenosine and its Receptors in Inflammation, Neuroinflammation, IBS, Autoimmune Inflammatory Disorders, Rheumatoid Arthritis and Psoriasis. Curr. Pharm. Des..

[138] Coppi E., Dettori I., Cherchi F., Bulli I., Venturini M., Pedata F., Pugliese A.M. (2020). New Insight into the Role of Adenosine in Demyelination, Stroke and Neuropathic Pain. Front. Pharmacol..

[139] Sawynok J., Reid A., Liu X.J. (2000). Involvement of mast cells, sensory afferents and sympathetic mechanisms in paw oedema induced by adenosine A_1_ and A_2B/3_ receptor agonists. Eur. J. Pharmacol..

[140] Green A., Milligan G., Belt S.E. (1991). Effects of prolonged treatment of adipocytes with PGE1, N6-phenylisopropyl adenosine and nicotinic acid on G-proteins and antilipolytic sensitivity. Biochem. Soc. Trans..

[141] Esquisatto L.C., Costa S.K., Camargo E.A., Ribela M.T., Brain S.D., de Nucci G., Antunes E. (2001). The plasma protein extravasation induced by adenosine and its analogues in the rat dorsal skin: Evidence for the involvement of capsaicin sensitive primary afferent neurones and mast cells. Br. J. Pharmacol..

[142] Yoon M.H., Bae H.B., Choi J.I., Jeong S.W., Chung S.S., Yoo K.Y., Jeong C.Y., Kim S.J., Chung S.T., Kim C.M. (2005). Evaluation of interaction between intrathecal adenosine and MK801 or NBQX in a rat formalin pain model. Pharmacology.

[143] Stemmer S.M., Benjaminov O., Medalia G., Ciuraru N.B., Silverman M.H., Bar-Yehuda S., Fishman S., Harpaz Z., Farbstein M., Cohen S. (2013). CF102 for the treatment of hepatocellular carcinoma: A phase I/II, open-label, dose-escalation study. Oncologist.

[144] Polosa R., Holgate S.T. (2006). Adenosine receptors as promising therapeutic targets for drug development in chronic airway inflammation. Curr. Drug Targets.

[145] Ramkumar V., Stiles G.L., Beaven M.A., Ali H. (1993). The A3 adenosine receptor is the unique adenosine receptor which facilitates release of allergic mediators in mast cells. J. Biol. Chem..

[146] Spruntulis L.M., Broadley K.J. (2001). A_3_ receptors mediate rapid inflammatory cell influx into the lungs of sensitized guinea-pigs. Clin. Exp. Allergy.

[147] Fossetta J., Jackson J., Deno G., Fan X., Du X.K., Bober L., Soudé-Bermejo A., de Bouteiller O., Caux C., Lunn C. (2003). Pharmacological analysis of calcium responses mediated by the human A_3_ adenosine receptor in monocyte-derived dendritic cells and recombinant cells. Mol. Pharmacol..

[148] Zylka M.J. (2011). Pain-relieving prospects for adenosine receptors and ectonucleotidases. Trends Mol. Med..

[149] Fedorova I.M., Jacobson M.A., Basile A., Jacobson K.A. (2003). Behavioral characterization of mice lacking the A_3_ adenosine receptor: Sensitivity to hypoxic neurodegeneration. Cell. Mol. Neurobiol..

[150] Yaar R., Lamperti E.D., Toselli P.A., Ravid K. (2002). Activity of the A_3_ adenosine receptor gene promoter in transgenic mice: Characterization of previously unidentified sites of expression. FEBS Lett..

[151] Carlin J.L., Jain S., Gizewski E., Wan T.C., Tosh D.K., Xiao C., Auchampach J.A., Jacobson K.A., Gavrilova O., Reitman M.L. (2017). Hypothermia in mouse is caused by adenosine A. Neuropharmacology.

[152] Yang J.N., Wang Y., Garcia-Roves P.M., Björnholm M., Fredholm B.B. (2010). Adenosine A_3_ receptors regulate heart rate, motor activity and body temperature. Acta Physiol..

[153] Baraldi P.G., Iaconinoto M.A., Moorman A.R., Carrion M.D., Cara C.L., Preti D., López O.C., Fruttarolo F., Tabrizi M.A., Romagnoli R. (2007). Allosteric enhancers for A_1_ adenosine receptor. Mini Rev. Med. Chem..

[154] Kiesman W.F., Elzein E., Zablocki J. (2009). A_1_ adenosine receptor antagonists, agonists, and allosteric enhancers. Handb. Exp. Pharmacol..

[155] Romagnoli R., Baraldi P.G., Carrion M.D., Cara C.L., Cruz-Lopez O., Salvador M.K., Preti D., Tabrizi M.A., Moorman A.R., Vincenzi F. (2012). Synthesis and biological evaluation of 2-amino-3-(4-chlorobenzoyl)-4-[(4-arylpiperazin-1-yl)methyl]-5-substituted-thiophenes. effect of the 5-modification on allosteric enhancer activity at the A_1_ adenosine receptor. J. Med. Chem..

[156] Giorgi I., Nieri P. (2013). Adenosine A_1_ modulators: A patent update (2008 to present). Expert Opin. Ther. Pat..

[157] Vincenzi F., Ravani A., Pasquini S., Merighi S., Gessi S., Romagnoli R., Baraldi P.G., Borea P.A., Varani K. (2016). Positive allosteric modulation of A. Neuropharmacology.

[158] Vincenzi F., Targa M., Romagnoli R., Merighi S., Gessi S., Baraldi P.G., Borea P.A., Varani K. (2014). TRR469, a potent A_1_ adenosine receptor allosteric modulator, exhibits anti-nociceptive properties in acute and neuropathic pain models in mice. Neuropharmacology.

[159] Ekblom A., Segerdahl M., Sollevi A. (1995). Adenosine increases the cutaneous heat pain threshold in healthy volunteers. Acta Anaesthesiol. Scand..

[160] Segerdahl M., Ekblom A., Sollevi A. (1994). The influence of adenosine, ketamine, and morphine on experimentally induced ischemic pain in healthy volunteers. Anesth. Analg..

[161] Rae C.P., Mansfield M.D., Dryden C., Kinsella J. (1999). Analgesic effect of adenosine on ischaemic pain in human volunteers. Br. J. Anaesth..

[162] Segerdahl M., Ekblom A., Sjölund K.F., Belfrage M., Forsberg C., Sollevi A. (1995). Systemic adenosine attenuates touch evoked allodynia induced by mustard oil in humans. Neuroreport.

[163] Sjölund K.F., Segerdahl M., Sollevi A. (1999). Adenosine reduces secondary hyperalgesia in two human models of cutaneous inflammatory pain. Anesth. Analg..

[164] Belfrage M., Sollevi A., Segerdahl M., Sjölund K.F., Hansson P. (1995). Systemic adenosine infusion alleviates spontaneous and stimulus evoked pain in patients with peripheral neuropathic pain. Anesth. Analg..

[165] Sjölund K.F., Belfrage M., Karlsten R., Segerdahl M., Arnér S., Gordh T., Solevi A. (2001). Systemic adenosine infusion reduces the area of tactile allodynia in neuropathic pain following peripheral nerve injury: A multi-centre, placebo-controlled study. Eur. J. Pain.

[166] Lynch M.E., Clark A.J., Sawynok J. (2003). Intravenous adenosine alleviates neuropathic pain: A double blind placebo controlled crossover trial using an enriched enrolment design. Pain.

[167] Sollevi A., Belfrage M., Lundeberg T., Segerdahl M., Hansson P. (1995). Systemic adenosine infusion: A new treatment modality to alleviate neuropathic pain. Pain.

[168] Hayashida M., Fukuda K., Fukunaga A. (2005). Clinical application of adenosine and ATP for pain control. J. Anesth..

[169] Moriyama M., Kitamura A., Ikezaki H., Nakanishi K., Kim C., Sakamoto A., Ogawa R. (2004). Systemic ATP infusion improves spontaneous pain and tactile allodynia, but not tactile hypesthesia, in patients with postherpetic neuralgia. J. Anesth..

[170] Segerdahl M., Ekblom A., Sandelin K., Wickman M., Sollevi A. (1995). Peroperative adenosine infusion reduces the requirements for isoflurane and postoperative analgesics. Anesth. Analg..

[171] Segerdahl M., Persson E., Ekblom A., Sollevi A. (1996). Peroperative adenosine infusion reduces isoflurane concentrations during general anesthesia for shoulder surgery. Acta Anaesthesiol. Scand..

[172] Segerdahl M., Irestedt L., Sollevi A. (1997). Antinociceptive effect of perioperative adenosine infusion in abdominal hysterectomy. Acta Anaesthesiol. Scand..

[173] Zárate E., Sá Rêgo M.M., White P.F., Duffy L., Shearer V.E., Griffin J.D., Whitten C.W. (1999). Comparison of adenosine and remifentanil infusions as adjuvants to desflurane anesthesia. Anesthesiology.

[174] Fukunaga A.F., Alexander G.E., Stark C.W. (2003). Characterization of the analgesic actions of adenosine: Comparison of adenosine and remifentanil infusions in patients undergoing major surgical procedures. Pain.

[175] Vincenzi F., Pasquini S., Battistello E., Merighi S., Gessi S., Borea P.A., Varani K.A. (2020). A_1_ Adenosine Receptor Partial Agonists and Allosteric Modulators: Advancing Toward the Clinic?. Front. Pharmacol..

[176] Rane K., Segerdahl M., Goiny M., Sollevi A. (1998). Intrathecal adenosine administration: A phase 1 clinical safety study in healthy volunteers, with additional evaluation of its influence on sensory thresholds and experimental pain. Anesthesiology.

[177] Eisenach J.C., Curry R., Hood D.D. (2002). Dose response of intrathecal adenosine in experimental pain and allodynia. Anesthesiology.

[178] Eisenach J.C., Hood D.D., Curry R. (2002). Phase I safety assessment of intrathecal injection of an American formulation of adenosine in humans. Anesthesiology.

[179] Eisenach J.C., Hood D.D., Curry R., Sawynok J., Yaksh T.L., Li X. (2004). Intrathecal but not intravenous opioids release adenosine from the spinal cord. J. Pain.

[180] Eisenach J.C., Rauck R.L., Curry R. (2003). Intrathecal, but not intravenous adenosine reduces allodynia in patients with neuropathic pain. Pain.

[181] Gaspardone A., Crea F., Tomai F., Iamele M., Crossman D.C., Pappagallo M., Versaci F., Chiariello L., Gioffrè P.A. (1994). Substance P potentiates the algogenic effects of intraarterial infusion of adenosine. J. Am. Coll. Cardiol..

[182] Sylvén C., Beermann B., Kaijser L., Jonzon B. (1990). Nicotine enhances angina pectoris-like chest pain and atrioventricular blockade provoked by intravenous bolus of adenosine in healthy volunteers. J. Cardiovasc. Pharmacol..

[183] Sawynok J., Reid A.R., Esser M.J. (1999). Peripheral antinociceptive action of amitriptyline in the rat formalin test: Involvement of adenosine. Pain.

[184] Ulugol A., Aslantas A., Ipci Y., Tuncer A., Hakan Karadag C., Dokmeci I. (2002). Combined systemic administration of morphine and magnesium sulfate attenuates pain-related behavior in mononeuropathic rats. Brain Res..

[185] Sandner-Kiesling A., Li X., Eisenach J.C. (2001). Morphine-induced spinal release of adenosine is reduced in neuropathic rats. Anesthesiology.

[186] Shapiro R.E. (2007). Caffeine and headaches. Neurol. Sci..

[187] Rao S.S., Mudipalli R.S., Remes-Troche J.M., Utech C.L., Zimmerman B. (2007). Theophylline improves esophageal chest pain--a randomized, placebo-controlled study. Am. J. Gastroenterol..

[188] Sebastião A.M., Cunha R.A., de Mendonça A., Ribeiro J.A. (2000). Modification of adenosine modulation of synaptic transmission in the hippocampus of aged rats. Br. J. Pharmacol..

[189] Correia-de-Sá P., Ribeiro J.A. (1994). Evidence that the presynaptic A_2a_-adenosine receptor of the rat motor nerve endings is positively coupled to adenylate cyclase. Naunyn-Schmiedeberg’s Arch. Pharmacol..

